# Supplementary material for: Influence of pneumatic transportation on the stability of monoclonal antibodies
Source: Sci Rep. 2023 Dec 10;13:21875. doi: 10.1038/s41598-023-49235-6 (PMC10710995; doi:10.1038/s41598-023-49235-6)
Supplement: Supplementary file 1 — Supplementary Figure 1. [file 41598_2023_49235_MOESM1_ESM.docx]

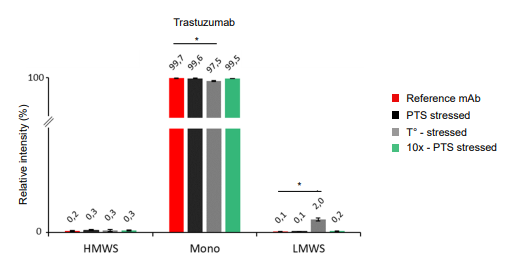


**Supplemental Figure 1 : Quantification of HMWS and LMWS from intact SEC-native MS analysis** **on trastuzumab**. Histograms represent SEC-UV peak area integration of aggregates (HMWS, mostly dimers) and fragments (mostly Fab-FC and Fab) for trastuzumab. *Red : Reference mAbs ; Black : 1 pass-PTS mAbs, Grey : Thermally stressed mAbs; Green: 10x pass-PTS mAbs.*
